# Environmental heterogeneity decreases reproductive success via effects on foraging behaviour

**DOI:** 10.1098/rspb.2019.0795

**Published:** 2019-06-05

**Authors:** Alice M. Trevail, Jonathan A. Green, Jonathan Sharples, Jeff A. Polton, Peter I. Miller, Francis Daunt, Ellie Owen, Mark Bolton, Kendrew Colhoun, Stephen Newton, Gail Robertson, Samantha C. Patrick

**Affiliations:** 1 School of Environmental Sciences, University of Liverpool, Liverpool, UK; 2 National Oceanography Centre, Liverpool, UK; 3 Remote Sensing Group, Plymouth Marine Laboratory, Plymouth, UK; 4 Centre for Ecology and Hydrology Edinburgh, Bush Estate, Penicuik, Midlothian, UK; 5 RSPB Centre for Conservation Science, RSPB Scotland, Etive House, Beechwood Park, Inverness, UK; 6 RSPB Centre for Conservation Science, The Lodge, Sandy, Bedfordshire, UK; 7 RSPB Centre for Conservation Science, Belfast, UK; 8 School of Agriculture and Food Science, University College Dublin, Bellfield, Dublin 4, Ireland; 9 BirdWatch Ireland, Kilcoole, Wicklow, Ireland; 10 School of Mathematics, University of Edinburgh, Edinburgh, UK

**Keywords:** competition, seabird, hidden Markov model, heterogeneity gradient, optimal foraging theory, resource availability

## Abstract

Environmental heterogeneity shapes the uneven distribution of resources available to foragers, and is ubiquitous in nature. Optimal foraging theory predicts that an animal's ability to exploit resource patches is key to foraging success. However, the potential fitness costs and benefits of foraging in a heterogeneous environment are difficult to measure empirically. Heterogeneity may provide higher-quality foraging opportunities, or alternatively could increase the cost of resource acquisition because of reduced patch density or increased competition. Here, we study the influence of physical environmental heterogeneity on behaviour and reproductive success of black-legged kittiwakes, *Rissa tridactyla*. From GPS tracking data at 15 colonies throughout their British and Irish range, we found that environments that were physically more heterogeneous were associated with longer trip duration, more time spent foraging while away from the colony, increased overlap of foraging areas between individuals and lower breeding success. These results suggest that there is greater competition between individuals for finite resources in more heterogeneous environments, which comes at a cost to reproduction. Resource hotspots are often considered beneficial, as individuals can learn to exploit them if sufficiently predictable. However, we demonstrate here that such fitness gains can be countered by greater competition in more heterogeneous environments.

## 1. Introduction

The spatial and temporal distribution of resources places a major constraint on foraging success [1–3]. Therefore, heterogeneity in resource distribution, which is considered a universal feature of natural environments [4,5], has played a defining role in the evolution of animal foraging behaviour [1,3]. Theory predicts that key to an individual's success is the ability to maximize gains from areas with high resource density and minimize energy expenditure locating resources, and therefore optimize energy allocation to fitness [2,3]. This theory is supported by numerous empirical studies (e.g. [6–8]). In response to resource heterogeneity, selection will therefore favour efficient foraging behaviour, whereby individuals minimize the energetic costs of searching and transiting between high-resource locations and maximize resource intake [1,3].

However, not all heterogeneous environments are equal [9–11], as high-prey locations vary in distribution, predictability and numbers of competing individuals. Studies often present these ‘prey hotspots’ as beneficial resource patches [12–14]; however, the optimality of foraging strategies in response to resource heterogeneity may be constrained by both the nature of resource heterogeneity [15,16] and the behaviour of other foragers [17,18]. First, the travel distance to reach foraging patches in heterogeneous environments will determine the trade-off between resource intake and the additional energetic costs to the animal's own fitness [16,19,20]. Second, higher levels of intraspecific competition at resource patches in heterogeneous environments may also limit resource acquisition from a patch [17,18,21,22] through competitive exclusion [18,22] and prey disturbance [17] and depletion [23]. The key knowledge gap is whether greater environmental heterogeneity has positive or negative consequences for fitness.

Underlying variability in the physical environment is a strong driver of heterogeneous resource distributions, and therefore can be used as a proxy for resource heterogeneity, particularly where resource availability to foragers is difficult to measure directly. Indeed, because of effects on resources, physical environmental heterogeneity, hereafter ‘environmental heterogeneity’, is known to be an important driver of community dynamics [11,24] and life-history strategies [25,26]. Marine environments provide a model study system of environmental heterogeneity, with numerous physical features (such as fronts, eddies and currents) that together define resource availability to foragers [12,27]. Furthermore, the degree to which any given marine environment is heterogeneous can vary [9], and therefore offers the opportunity to study the influence of heterogeneity on behaviour and fitness.

In this study, we test the influence of environmental heterogeneity on behaviour and reproductive success using data from black-legged kittiwakes (*Rissa tridactyla*, hereafter ‘kittiwakes’) at 15 colonies across their UK and Irish breeding range. Studying such a comprehensive dataset is ideal to understand how environmental heterogeneity affects behaviour and fitness. As with many seabirds, kittiwakes are central place foragers during the breeding season, and are therefore constrained to forage within their local environment. As such, greater travel distances away from the breeding location are considered indicative of poorer resource availability nearby [28,29]. Furthermore, as surface feeders, kittiwakes are thought to suffer from direct competition with conspecifics for prey as fish schools are forced lower down in the water column to inaccessible depths [30,31]. We first calculate a measure of local environmental heterogeneity at each colony based on six environmental metrics that can all influence kittiwake prey distributions. Second, we consider kittiwake foraging behaviour along the gradient of environmental heterogeneity between study colonies, and then test the link between the degree of environmental heterogeneity and reproductive success. Our analyses tested the following alternative hypotheses (see table 1) based on the literature reviewed above. (H1) Foraging opportunity hypothesis: greater environmental heterogeneity is associated with higher fitness because it features greater amounts of profitable habitat within the foraging range of the colony that animals can learn to exploit, which enables individuals to remain closer to the colony [16], provision offspring more frequently [32] and relieve partners of nest-attendance duties [33]. (H2) Reduced patch density hypothesis: greater environmental heterogeneity is detrimental to fitness because habitat patches with sufficient resources to support foraging are located further apart within the foraging range. This, therefore, would prompt individuals to more readily switch between patches [2], requiring an increase in travel distance away from the colony, time spent commuting and foraging area size [16], and resulting in greater expenditure to transit between patches. (H3) Competition hypothesis: greater environmental heterogeneity is detrimental to fitness because it increases competition between individuals at relatively profitable habitats, which results in greater overlap between individuals, greater time investment in foraging behaviour and increased duration of foraging trips [17].

**Table 1.** Hypotheses of the potential effects of greater environmental heterogeneity on resources, foraging dynamics and fitness at the population level. Upwards and downwards arrows indicate an expected increase and decrease, respectively, and crosses indicate no expected change.

			expected change in foraging metrics			
hypothesis	potential effect of greater environmental heterogeneity on resources	expected change in population foraging dynamics	travel distance (maximum, total and proportion of time transiting)	time foraging (trip duration and proportion of time foraging)	competition (overlap between individuals)	expected change in fitness
(H1) foraging opportunity	resource patches present higher quality foraging opportunities	smaller foraging range 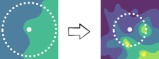	↓	↓	X	↑
(H2) reduced patch density	resource patches offering sufficient foraging opportunities are further apart	longer foraging distance 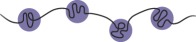	↑	↑ or X	X or ↓	↓
(H3) competition	resources concentrated into smaller patches	increased competition 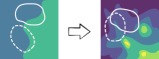	X	↑	↑	↓

## 2. Methods

### (a) Quantifying environmental heterogeneity

To quantify environmental heterogeneity, we used a multivariate dispersion analysis [34] to identify the dissimilarity of spatial environmental characteristics. Multivariate dispersion analyses have primarily been used for species diversity studies [34]; however, they have also been used to quantify environmental heterogeneity using multiple continuous variables in studies of freshwater ecosystems [35,36], marine ecosystems [34] and grasslands [37]. Multivariate dispersion analysis is suitable for this study because it incorporates variance in multiple environmental parameters that can all influence resource distribution into a single metric, in contrast to measures such as standard deviation or range of a single continuous variable [10,38], or the diversity of categorical habitat variables [39].

We calculated environmental heterogeneity using six environmental metrics: (1) bathymetry, (2) potential tidal stratification, (3) sea surface temperature, and ocean front (4) strength, (5) distance and (6) persistence, all of which have been shown to influence resource location for foraging seabirds. (1) Bathymetry, or sea floor depth, can shape the flow of horizontal water currents and control vertical water column structure [40,41], both of which are physical processes that can influence the availability and accessibility of prey fish to surface foragers such as kittiwakes [27,40,41]. (2) Potential tidal stratification incorporates both depth and tidal currents [12], to quantify the vertical water column structure—a key physical driver of marine ecosystem dynamics [42], prey fish distribution [43] and seabird distribution [27]. (3) Sea surface temperature can be a proxy for oceanographic processes that influence nutrient availability, such as upwelling of cold nutrient-rich water [44], and has been linked to the at-sea distribution and breeding success of kittiwakes [27,42,45,46]. (4–6) Ocean fronts are horizontal boundaries between different water masses where physical processes cause upwelling of deeper, nutrient-rich water and entrain plankton at the surface [47,48]. Fronts are known to be an important feature of marine environments, shaping resource distribution and thus marine vertebrate behaviour [43,49]. Full details of data sources are described in electronic supplementary material, appendix A.

We used a principal coordinate analysis (a type of multivariate dispersion analysis) [50] to determine the heterogeneity of environmental conditions at each colony and year (hereafter ‘colony-year’) from within the maximum foraging range of kittiwakes. We used the overall maximum foraging range across all years as a measure of the environment available to each colony (electronic supplementary material, appendix B). Principal coordinate analyses place values from all colonies along all axes (or principal coordinates) in unconstrained ordination space based on a Euclidean distance matrix of standardized environmental data, using the functions *vegdist* and *betadisper* in the R package *vegan* [51]. Herein, we use the average distance of observations from the colony-year centroid (or spatial median) in the principal coordinate analysis ordination space (using all axes) as a continuous measure of environmental heterogeneity, with higher values indicating greater heterogeneity. As such, environmental heterogeneity can vary independently of the absolute values of the six environmental variables. Permutation tests of dispersion (PERMDISP [34]) calculate an *F-statistic* to compare the average distances of observations from the colony-year centroid between each colony-year in the analysis to test for differences in heterogeneity. We used a two-way ANOVA to test whether environmental heterogeneity differed between colonies and between years (as factors), and Tukey HSD *post hoc* tests for pairwise differences. To understand whether environmental heterogeneity was simply associated with availability of a particular habitat type or was a proxy of overall prey abundance within the foraging range of kittiwakes (maximum foraging distance across years at each colony from tracking data; H1 and H2, table 1), we used linear regression to test whether environmental heterogeneity was linked to the mean value of any of the individual environmental metrics. To determine whether environmental heterogeneity was influenced by the size of the foraging radius used to extract environmental data (maximum foraging distance across years at each colony), we compared environmental heterogeneity values with the maximum foraging range of kittiwakes at each colony across all years using linear regression.

### (b) Quantifying kittiwake foraging behaviour

**Figure 1.**
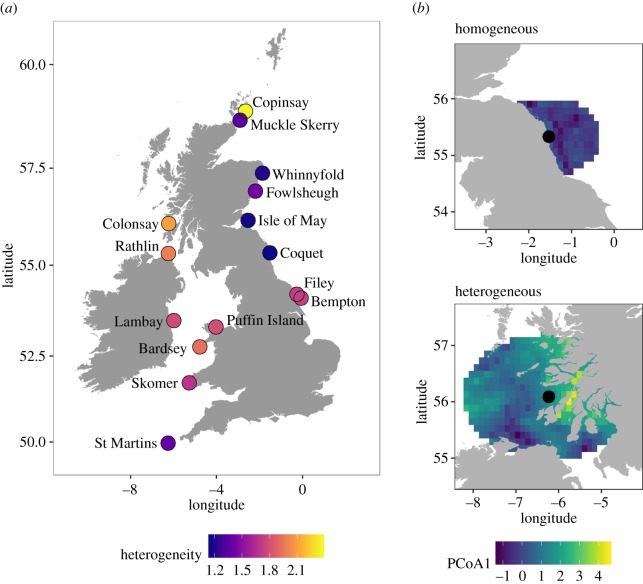
(*a*) Map of study kittiwake colonies, coloured by mean environmental heterogeneity and (*b*) environment within the foraging range of two example colonies according to the position along the first axis (PCoA1) from the principal coordinate analysis used to calculate environmental heterogeneity, here for 2015 as an example. Colony environmental heterogeneity is a single measure of variance calculated as the mean distance in Euclidian space (using all PCoA axes) of all locations from the colony centroid. At the homogeneous colony (Coquet, top), values are concentrated together along the first PCoA axis. At the heterogeneous colony (Colonsay, bottom), values range along the first PCoA axis. (Online version in colour.)

To determine the foraging behaviour of kittiwakes around the UK, adults from multiple colonies were tracked using GPS loggers (Mobile Action i-GotU GT-120), while raising small chicks. Tracked individuals were selected randomly with respect to brood size and were assumed to be representative of each study population. Loggers were attached to the back feathers between the wings (or infrequently to the tail) using waterproof tape, and total instrument mass was less than or equal to 5% of body mass (or less than or equal to 3% where tail attachments were used; mean ± s.e. body mass at Skomer, Rathlin and Puffin Island: 327.9 ± 5.1 from Trevail *et al*. [9]). Full details of tracking procedures can be found in the first publications of the data: Wakefield *et al*. [27] and Trevail *et al*. [9]. Here, we use data from a total of 1567 trips from 415 chick-rearing kittiwakes at 15 colonies in Britain and Ireland between 2010 and 2017 (figure 1): Bardsey (NW Wales; 2011, *n* = 8), Bempton Cliffs (E England; 2010–2013 and 2015, *n* = 59), Copinsay (Orkney Islands; 2010–2012, *n* = 26), Coquet (NE England; 2011–2012, *n* = 26), Colonsay (W Scotland; 2010–2014, *n* = 69), Filey (E England; 2013 and 2015, *n* = 26), Fowlsheugh (E Scotland; 2012, *n* = 13), Isle of May (E Scotland; 2013, *n* = 16), Lambay (E Ireland; 2010, *n* = 10), Muckle Skerry (Orkney Islands; 2012–2014, *n* = 26), Puffin Island (NW Wales; 2010–2016, *n* = 63), Rathlin (Northern Ireland; 2017, *n* = 17), Skomer (SW Wales; 2016–2017, *n* = 14), St Martins (Isles of Scilly; 2010–2011, *n* = 28) and Whinnyfold (E Scotland; 2012, *n* = 14). Full sample sizes, including colony coordinates, tracking dates and number of individuals per year are given in electronic supplementary material, table B1. For further analyses, we excluded points closer than 500 m to the colony, and attributed sequential points to a foraging trip if the total trip duration was over 14 min [9] to eliminate departures from the colony due to disturbance [52]. At all colonies, we included trips where individuals were away from the colony overnight. At Rathlin, Skomer and Puffin Island, loggers did not record data between 23.00 and 03.00, to save battery power overnight while kittiwakes exhibit minimal foraging activity [9,53]. At all other colonies, we excluded locations during this period.

To understand the influence of environmental heterogeneity on foraging behaviour, we calculated three different measures of behaviour, all predicted to vary with each hypothesis (table 1). First, for each year and at each colony, we calculated the following trip metrics: mean trip duration, mean total distance travelled during a foraging trip, and mean maximum distance from the colony, all important indicators of resource accessibility for central place foragers as they seek to remain close to the colony and minimize travel times [16,17,28]. Second, we examined movement behaviours while away from the colony using a hidden Markov model to classify behaviour into rest, forage (including searching) or transit [54]. Time spent in each behaviour can signal the energetic trade-off between travel costs and resource gains from exploiting prey patches [55]. We used the R package moveHMM [56] for behavioural classification based on distributions of step lengths and turning angles, after interpolating GPS data to regular time steps to fulfil HMM assumptions, using the R package adehabitatLT [57]. We used a gamma distribution to describe step lengths and a von Mises distribution to describe turning angles, and the Viterbi algorithm to estimate the most likely sequence of movement states based on the fitted hidden Markov model (electronic supplementary material, appendix C). We used values from the previous classification of kittiwake behaviour to inform model starting parameters [9], and found that model outputs were robust to different values of starting parameters when tested on a subset of tracking data. For each bird, we quantified the proportion of time away from the colony while on a foraging trip spent in each behaviour classified by the HMM (forage, transit and rest). Third, we determined at-sea area use of kittiwakes by calculating the size of 50% core foraging areas of individuals from utilization kernels on a 1 km grid using the *kernelUD* function in the R package adehabitatHR [57]. The appropriate smoothing parameter (*h*) was determined by the default ad hoc method, which assumes a bivariate normal distribution [57]. As a proxy for intra-specific competition, we calculated the overlap of 50% core foraging areas between all individuals tracked in the same year at each colony using Bhattacharya's affinity (BA). Values of BA range from 0 when there is no overlap between foraging areas to 1 when utilization distributions are identical [58].

### (c) Quantifying kittiwake reproductive success

To test the effect of environmental heterogeneity on kittiwake reproductive success, we used colony-average reproductive success data from the Seabird Monitoring Programme, collated by the UK Joint Nature Conservation Committee (JNCC; http://jncc.defra.gov.uk/smp) and the Centre for Ecology & Hydrology for the Isle of May [59]. Reproductive success data were available for 11 colonies, for 1–8 years between 2010 and 2017 (electronic supplementary material, table B3). Reproductive success was calculated as the total number of chicks fledged divided by the number of nests/pairs monitored at each colony in each year (electronic supplementary material, table B4 and figure B1).

### (d) Effect of environmental heterogeneity on kittiwake foraging behaviour and reproductive success

In all analyses described below, explanatory variables were standardized to a mean of 0 and standard deviation of 1. Model structure and effect significance were determined using ANOVA comparisons (χ^2^ for linear regressions and GLMMs, and F tests for quasi-binomial), for which *p*-values are presented.

To understand the effects of environmental heterogeneity on foraging metrics and reproductive success, we used the mean environmental heterogeneity for each colony across all years because colony and year, by definition, explained a large proportion of the variation in environmental heterogeneity (electronic supplementary material, figure A3), and did not include colony or year as variables in regression analyses. We refer to this mean value as ‘colony-mean environmental heterogeneity’. To understand the effect of environmental heterogeneity on foraging behaviour in relation to the hypotheses (table 1), we undertook the following statistical tests. First, we compared the colony-mean environmental heterogeneity to the annual mean of trip metrics for each colony (trip duration, total distance and maximum distance; log-transformed to meet the assumptions of Gaussian models) using linear regression. Second, we compared the colony-mean environmental heterogeneity to the proportion of time spent away from the colony in each behavioural state (forage, transit and rest) by each individual using linear regression with a quasi-binomial logit-link to account for overdispersion. Lastly, we compared the colony-mean environmental heterogeneity with the size of 50% core foraging area of each bird using linear regression, and overlap between trips of all pairs of individuals using a GLMM with the focal BirdID as a random effect and a Gaussian distribution. To understand the effect of environmental heterogeneity on reproductive success, we compared the colony-mean environmental heterogeneity with the annual reproductive success for each colony using linear regression.

To verify that observed patterns in foraging dynamics and resource success could be attributed to environmental heterogeneity, we tested for potentially confounding effects of colony size and individual environmental variables on reproductive success (electronic supplementary material, appendix D). We used data from the most recent census of UK breeding populations, Seabird 2000 [60], to compare breeding success with colony size and the number of breeding kittiwakes within the foraging radius of each colony using linear regression. Seabird 2000 data may no longer provide currently accurate estimates of breeding numbers; however, they offer the most useful indicator of relative colony size for the purpose of this study. In support of results presented below, we found no link between reproductive success and any environmental metric in isolation (bathymetry, stratification, sea surface temperature and ocean front metrics; electronic supplementary material, table D1), suggesting that heterogeneity in resource distribution is key in this system.

## 3. Results

### (a) Environmental heterogeneity at colonies

Environmental heterogeneity varied significantly between colony and year combinations in the principal coordinate analysis (figure 1; *F*_119,17880_ = 16.6, *p* < 0.001). The first two coordinate axes from the principal coordinate analysis together explained 63% of the total variation between colonies (first axis: 43.1%, all others presented in electronic supplementary material, table A1). Environmental heterogeneity differed significantly between colonies (figure 1; ANOVA: *F*_14,98_ = 42.8, *p* < 0.001), and between years (ANOVA: *F*_7,98_ = 3.0, *p* = 0.007), although the effect of year was driven by a significant difference between 2011 and 2014 (electronic supplementary material, appendix A). Environmental heterogeneity was highest at Copinsay (mean ± s.e. between years = 2.37 ± 0.05), and was lowest at Coquet (1.16 ± 0.06), the Isle of May (1.17 ± 0.04) and Whinnyfold (1.19 ± 0.07). Values of environmental heterogeneity at each colony, and pairwise comparisons between colonies and years are given in electronic supplementary material, appendix A. Comparisons of environmental heterogeneity with individual environmental metrics showed no strong relationships (electronic supplementary material, appendix E). There was no link between environmental heterogeneity from the principal coordinate analysis and the size of the radius (maximum foraging distance from the colony across all years) used to select environmental data (*F*_(1,118)_ = 0.76, *p* = 0.386).

### (b) Hypothesis testing: effect of environmental heterogeneity on kittiwake foraging behaviour and reproductive success

**Figure 2.**
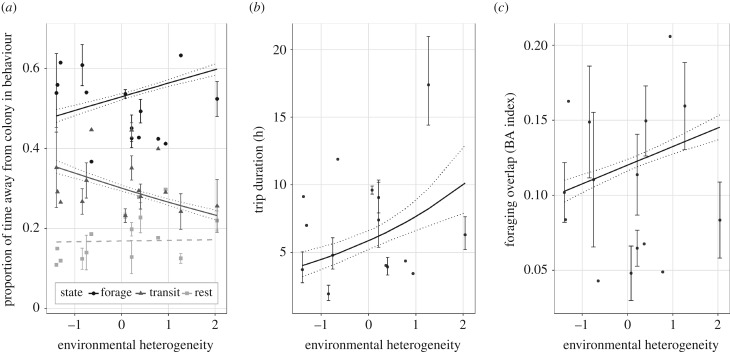
Comparison between environmental heterogeneity and foraging behaviour of kittiwakes. (*a*) The proportion of individuals' time while away from the colony in different behavioural states varied over the observed range of heterogeneity: time spent foraging significantly increased (*F*_1,415_ = 18.8, *p* < 0.01), time spent transiting significantly decreased (*F*_1,415_ = 23.5, *p* < 0.01), and there was no change in time spent resting (dashed line, *F*_1 ,415_ = 0.08, *p* = 0.78). (*b*) Trip duration significantly increased over observed range of heterogeneity (*F*_1,33_ = 5.11, *p* = 0.031). (*c*) Overlap between pairs of individuals' 50% core foraging areas significantly increased over observed range of heterogeneity ($\chi_{1}^{2}=9.85,$ *p* = 0.002). Colony environmental heterogeneity is a measure of variance using a principal coordinate analysis. In all cases, error bars show standard error around the mean where GPS data were collected in multiple years, and solid lines show significant regressions with standard error (dotted lines).

**Figure 3.**
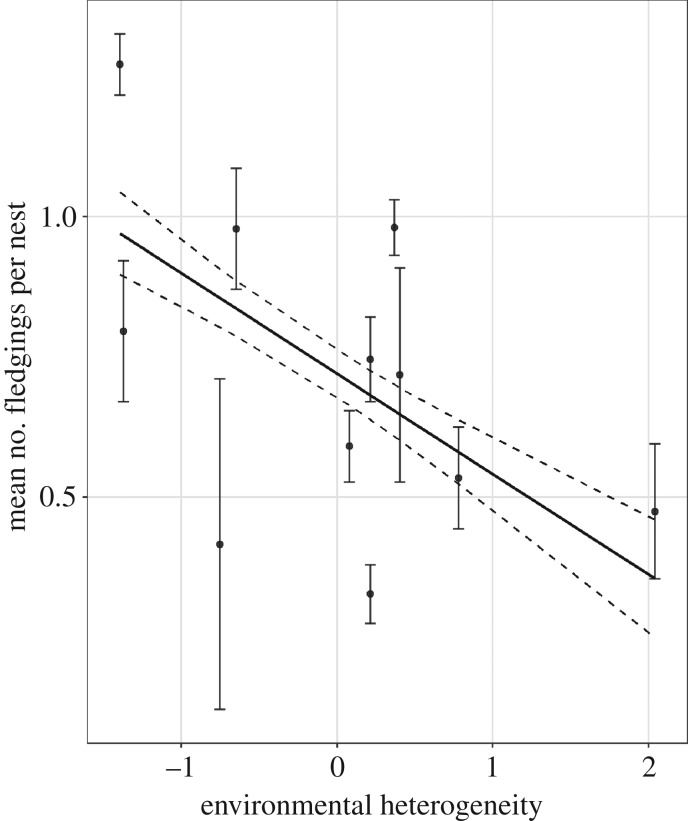
Kittiwake reproductive success compared with standardized environmental heterogeneity. Solid line shows a significant regression ± s.e. (dashed lines) between environmental heterogeneity and reproductive success (*F*_1,59_ = 15.44, *p* < 0.001, *R*^2^ = 0.21). Colony environmental heterogeneity is a measure of variance using a principal coordinate analysis. Error bars show standard error around the mean reproductive success from multiple years.

We found most support for the competition hypothesis (H3) that environmental heterogeneity was associated with greater competition between individuals, and consequently lower fitness. We found that trip duration (time spent away from the colony) was positively correlated with environmental heterogeneity (figure 2*b*; parameter estimate ± s.e.: 0.27 ± 0.12, *F*_1,33_ = 5.11, *p* = 0.03). Furthermore, the proportion of individuals' time spent foraging was significantly higher in more heterogeneous environments (figure 2*a*; parameter estimate ± s.e.: 0.14 ± 0.03 *F*_1,415_ = 18.8, *p* < 0.01), and environmental heterogeneity was positively correlated with overlap of the 50% core foraging area between individuals (figure 2*c*; parameter estimate ± s.e.: 0.012 ± 0.004, $\chi_{1}^{2}=9.85,$ *p* < 0.01). Reproductive success was significantly lower in colonies with greater environmental heterogeneity (figure 3; parameter estimate ± s.e. = −0.18 ± 0.05; *F*_1,59_ = 15.44, *p* < 0.01), equivalent to a 63% decrease in reproductive success across the observed range of environmental heterogeneity. This relationship is robust to removal of the apparent outlier of Copinsay. We did not find support for the foraging opportunity hypothesis (H1) that environmental heterogeneity was associated with greater amounts of profitable habitat: there was no link between environmental heterogeneity and the mean maximum distance kittiwakes travelled from the colony (table 2; *F*_1,33_ = 1.11, *p* = 0.30). Lastly, we did not find support for the reduced patch density hypothesis (H2) that environmental heterogeneity is associated with greater distances between relatively profitable foraging areas, since there was no link between environmental heterogeneity and the mean maximum distance travelled (detailed above) or the total distance travelled (table 2; *F*_1,33_ = 2.59, *p* = 0.12). The proportion of individuals’ time spent transiting was significantly lower in more heterogeneous environments (figure 2*a*; parameter estimate ± s.e.: −0.17 ± 0.04, *F*_1,415_ = 23.5, *p* < 0.01), and there was no change in the time spent resting (figure 2*a*; *F*_1,415_ = 0.08, *p* = 0.78). There was no link between environmental heterogeneity and the size of an individual's 50% core foraging area (table 2; *F*_1,414_ = 0.34, *p* = 0.56). In support of the above results that environmental heterogeneity is an important mechanism driving fitness, reproductive success was not linked to colony size (*F*_1,51_ = 0.96, *p* = 0.33) or the number of kittiwakes breeding within the foraging radius of the colony (*F*_1,59_ = 1.64, *p* = 0.21).

**Table 2.** Changes in kittiwake behaviour and reproductive success over the range of environmental heterogeneity observed in this study. Rows in italic type showed a significant relationship (*p* < 0.05).

response variable	relationship with increasing heterogeneity	parameter estimate	units	test statistic	*p*-value
*proportion of time foraging*	*increase*	*0.09* *± 0.04*	*proportion*	*F_(1,415)_ = 18.8*	*p = 0.029*
*proportion of time transiting*	*decrease*	*−0.17 ± 0.04*	*proportion*	*F_(1,415)_ = 23.5*	*p < 0.001*
proportion of time resting	no difference	0.04 ± 0.05	proportion	*F*_(1,415)_ = 0.08	*p* = 0.479
*mean trip duration*	*increase*	*0.27* *± 0.12*	*hours (log-scale)*	*F_(1,33)_ = 5.11*	*p = 0.031*
mean total distance	no difference	0.19 ± 0.12	km (log-scale)	*F*_(1,33)_ = 2.59	*p* = 0.117
mean maximum distance	no difference	0.12 ± 0.11	km (log-scale)	*F*_(1,33)_ = 1.11	*p* = 0.299
*foraging area: overlap*	*increase*	*0.01* *± 0.00*	*BA index*	$\chi_{1}^{\;2}\;=9.85$	*p = 0.002*
foraging area: size	no difference	42.2 ± 71.7	km^2^	*F*_(1,414)_ = 0.34	*p* = 0.561
*breeding success*	*decrease*	*−0.18 ± 0.05*	*fledglings per nest*	*F_(1,59)_ = 15.4*	*p < 0.001*

## 4. Discussion

Heterogeneous resources are inherent within nature [4,5,61], and are typically assumed to be beneficial to foragers [12,13,62]. However, our study demonstrates that in areas of higher environmental heterogeneity (or greater patchiness), kittiwakes undertook longer foraging trips, spent proportionally more time foraging while away from the colony, overlapped more with other individuals and had reduced breeding success. Together, these results are consistent with our hypothesis that environmental heterogeneity may have concentrated resources into relatively more profitable patches; however, this resulted in greater intraspecific competition, with negative consequences for fitness.

Heterogeneous environments can concentrate resources into patches that animals can adapt their behaviour to, in theory to optimize foraging efficiency [1,63]. Indeed, here we show differences in foraging behaviour with environmental heterogeneity; specifically, in more heterogeneous environments kittiwakes undertook longer foraging trips, and while away from the colony spent more time foraging. If overall resource availability was higher in heterogeneous environments, such changes in foraging behaviour could be an adaption to increase resource acquisition. However, by contrast, we found that reproductive success was lower in heterogeneous environments, suggesting that greater time investment in foraging behaviour was not compensated for by higher energetic returns [64]. Furthermore, we show that in colonies with more heterogeneous local environments, pairs of individuals overlapped more in their core foraging areas, despite no difference in individual foraging area size. These results suggest that in more heterogeneous environments there is more competition between individuals for finite resources, with costs for reproductive success. While this may be balanced by lower competition elsewhere, lower resource availability away from resource patches will limit resource gains, and where resources are concentrated, resource density may still not be sufficient to benefit all competing individuals. Increased competition between individuals also explains extended foraging trip duration, as acquiring sufficient resources takes more time [17,31], which could incur additional energetic costs on adults, reduce offspring provisioning rates and increase the risk of offspring predation during brood neglect [17,18]. Bio-logging devices can cause a slight increase in trip duration [65]; however, we would expect such effects to be equal across colonies. As such, fitness gains from resource patches may in fact be limited by the degree of environmental heterogeneity, because of the potential cost of competition.

Environmental heterogeneity may also decrease reproductive success if a greater variability of habitat types reduces the amount of productive habitat and/or is associated with generally lower primary productivity. If that were the case, we would expect foragers in heterogeneous environments to have to travel further from the colony in order to access high-quality habitat [16,29,66,67]. However, we found no difference in how far kittiwakes travelled away from the colony in heterogeneous environments, even accounting for the size of breeding populations. Maximum foraging distances recorded here (mean maximum distance: 23.3 ± 0.8 km) were within both theoretical and observed ranges of the species (e.g. theoretical based on Isle of May data and kittiwake flight speeds: 73 ± 9 km [27,53], observed at Pribilof Islands, Bering Sea, Alaska: 206.7 ± 6.7 km [68] and observed at Sør-Gjæslingan, Norway: 303.7 ± 6.1 km [69]). We can therefore assume that individuals were not foraging at, or near, their maximum physiological capability, but rather that sufficient resource availability facilitated individuals to remain within relative proximity of the colony. Alternatively, heterogeneity may decrease reproductive success if profitable resource patches are more dispersed in space [18], requiring greater travel distances to reach sufficient resource patches [16]. However, we found no difference with environmental heterogeneity in the total distance travelled during a foraging trip, and no increase in the proportion of a trip spent transiting or the size of an individual's 50% core foraging area, suggesting no increase in space use to acquire resources. Heterogeneous environments may, however, require behavioural adaptations that, if not compensated for by energetic gains, could contribute to the reduced breeding success observed in this study [70]. For example, environmental heterogeneity can drive the magnitude of temporal variability in resources, which in turn prompts a greater behavioural response to temporal cycles in heterogeneous environments [9].

Foraging behaviour (in particular, foraging range) is typically linked to colony size in central place breeders. Density-dependent prey depletion can increase the colony foraging radius [29,67], up to the physiological constraints of a species, which can then limit the carrying capacity [66]. As such, when considering foraging adaptations and reproductive consequences of environmental heterogeneity here, it is important to recognize the potential effect of colony size. However, we found no link between reproductive success and colony size, nor the number of kittiwakes breeding within the foraging range of the colony, in contrast to previous studies of seabird population dynamics [31]. Our results therefore suggest that the spatial distribution of resources, as shaped by environmental heterogeneity, could be the predominant mechanism driving differences in levels of intraspecific competition, and therefore reproductive success, between kittiwake colonies in the UK and Ireland. Colony size data were from the most recent full census of the UK and Ireland seabird breeding colonies in 2000 [60]. Many sites have documented population declines both before and after the Seabird 2000 survey, and kittiwakes have been reclassified as vulnerable on the IUCN Red List of threatened species [71]. It is therefore likely that during the years of this study, population numbers were well below historic carrying capacity; however, the Seabird 2000 data provide a useful indication of population numbers for this study.

The degree of environmental heterogeneity at each colony remained relatively consistent over time, which may favour an individual to switch breeding colony in favour of homogeneous sites where reproductive success was higher [72,73]. Reproductive success was, however, generally low; at all but one colony in this study (Coquet) kittiwakes reared less than one fledgling per nest on average. This may mean that the potential increase in reproductive success in more homogeneous environments is not worth the risk of switching breeding site, but instead is outweighed by other factors driving strong site fidelity common among seabirds such as pair bonds [74], familiarity with conspecifics [75] and natal philopatry [76]. Future study could, however, shed light on the effect of environmental heterogeneity on recruitment of prospecting breeders, as well as long-term population trends [77].

## 5. Conclusion

In this study, we demonstrate that, in contrast to common assumptions, environmental heterogeneity is detrimental to breeding success in this species. Environmental heterogeneity can concentrate resources into hotspots, which could offer foraging opportunities; however, it may also increase competition between individuals. Reproductive success is an important driver of population dynamics across taxa [78,79], including adult recruitment in kittiwakes [77], and therefore the results of this study highlight the potential importance of environmental heterogeneity for driving population success and species distributions. Furthermore, environmental heterogeneity may be a key consideration in future studies of species resilience to environmental stressors, particularly given that many species, including kittiwakes, are undergoing population declines.

## Supplementary Material

Electronic supplementary material

## Data accessibility

Data and code used for analyses are available from the Dryad Digital Repository: https://doi.org/10.5061/dryad.0898ss6 [80] and were made available to editors and reviewers upon submission. Kittiwake tracking data can be accessed via the BirdLife International Seabird Tracking Database.
