# Supplementary material for: Environmental heterogeneity decreases reproductive success via effects on foraging behaviour
Source: Proc Biol Sci. 2019 Jun 5;286(1904):20190795. doi: 10.1098/rspb.2019.0795 (PMC6571457; doi:10.1098/rspb.2019.0795)
Supplement: Electronic supplementary material [file rspb20190795supp1.docx]

Supplementary Material to:

**Environmental heterogeneity decreases reproductive success via effects on foraging behaviour**

Appendix A. Environmental heterogeneity at study colonies and between years

Appendix B: GPS tracking sample sizes & summary trip metrics

Appendix C: Hidden Markov models for behavioural classification

Appendix D: Other environmental variables

Appendix E: Comparison of environmental heterogeneity with foraging range and environmental variables

References

**Appendix A. Environmental heterogeneity at study colonies and between years**

In order to calculate environmental heterogeneity, we used the six environmental variables described below, (1) bathymetry, (2) potential tidal stratification, (3) sea surface temperature, and ocean front (4) strength, (5) distance and (6) persistence. We averaged all dynamic spatial variables (sst, front density, front persistence and front distance) over June and July at each colony for each year to incorporate all tracking dates and match the temporal resolution of foraging metrics and reproductive success. We found good covariance between environmental heterogeneity calculated using seasonally averaged environmental data and environmental heterogeneity calculated using weekly measures of environment variables (Figure A1). To format spatial environmental data for analyses, we first resampled all environment data to the same resolution, and then randomly selected 150 points using the *sampleRandom* function of the R package *raster* [1] from within the maximum foraging range of kittiwakes (linear distance from the colony) at each colony in order to achieve a balanced design for post hoc analyses. We extracted environment data from within the maximum foraging range of kittiwakes at each colony across all years to represent the available environment at each colony. Considering the potential effect of sample size to affect the observed foraging range [2], we only included years with six or more individuals tracked at a colony in all analyses in this study. In support of using the colony-maximum foraging radius to calculate environmental heterogeneity, we found no effect of the number of GPS tracking data years on the size of the colony-maximum foraging radius (F_(1,13)_=2.73, p = 0.123).

*(1) Bathymetry,* or depths of the sea floor, can shape water currents and control the vertical structure of the water column, and is known to influence kittiwake behaviour [3]. Bathymetric data were collated from high resolution UK Hydrographic Office (UKHO) Data Archive Centre for bathymetric surveys and integrated with the 2014 General Bathymetric Chart of the Oceans (GEBCO). UKHO data contains public sector information, available under the Open Government Licence as part of the INSPIRE initiative. UKHO data, collected from boat surveys, covered a subset of the study areas at between 2 and 4 m resolution.

*(2) Potential tidal stratification* is an index for vertical water column structure in areas shallower than 200 m [4]. Low values of potential tidal stratification indicate a typically well-mixed water column. High values indicate a typically more stratified water column, comprised of vertically distinct layers with reduced transfer of abiotic and biotic matter between layers. Potential tidal stratification is calculated as log_10_(h/U^3^), where *h* is water column depth, described above, and *U* is the sum of tidal amplitude from both the M2 (semidiurnal lunar tide) and S2 (semidiurnal solar tide) tidal constituents [5]. Tidal amplitudes were generated from a 3D hydrostatic simulation of the North West European shelf using the NEMO AMM60 configuration [4]. The simulation has 51 stretched layers in the vertical and a resolution of 1.8 km in the horizontal. A barotropic harmonic analysis was performed on the simulation and the M2 constituent is processed here (as the most energetic constituent). The M2 tidal speeds are defined as the amplitude of maximum barotropic M2 velocity, over the tidal cycle. Potential tidal stratification performs well as a predictor of seasonal thermal stratification in shelf sea areas, although interpretation of absolute values requires caution in regions where stratification is influenced by salinity as a result of freshwater riverine inputs [6].

*(3) Sea surface temperature* (SST) can both indicate oceanographic processes that influence water column structure, such as upwelling of colder water [7], and can be a proxy for the quality of prey availability [8]. SST data were derived from seven-day composites of advanced very high-resolution radiometer data, at a resolution of approximately 1.2 km (AVHRR).

*(4-6) Oceanic fronts* are horizontal boundaries between different water masses where resource availability is typically enhanced because of physical processes that cause upwelling of deeper, nutrient rich water. Fronts are therefore known to be an important feature of marine environments, shaping resource distribution and thus marine vertebrate behaviour [9,10]. Frontal contours were detected using local regional statistics on daily SST scenes from AVHRR satellite data, at a resolution of approximately 1.2 km, and then combined into 8-day composite front maps [11]. Here we include three parameters related to ocean fronts, all of which will influence resource availability to a central place forager: (1) Front density gives the mean thermal gradient magnitude of detected fronts, i.e. the strength of fronts, spatially smoothed to give a continuous distribution of frontal activity. (2) Front distance indicates the distance at each pixel to the closest major front, determined using a simplified version of the frontal strength map. (3) Front persistence indicates the fraction of cloud-free observations of a pixel for which a front was detected, i.e. whether fronts are temporally persistent or only short-lasting, spatially smoothed to give a continuous distribution.

Table A1. Eigenvalues from Principal Coordinate analyses of environmental variables used to calculate environmental heterogeneity, and cumulative explained variance. All axes are used to calculate environmental heterogeneity as the average distance of each observation from the colony-year centroid in ordination space.

| Axis | Eigenvalue | Cumulative variance explained (%) |
| --- | --- | --- |
| PCoA1 | 46540 | 43.1 |
| PCoA2 | 21538 | 63.0 |
| PCoA3 | 17282 | 79.0 |
| PCoA4 | 11658 | 89.8 |
| PCoA5 | 8988 | 98.2 |
| PCoA6 | 1987 | 100.0 |

Table A2. Environmental heterogeneity, calculated using a principal coordinate analysis of six environmental variables (bathymetry, potential tidal stratification, sea surface temperature, and ocean front strength, distance and persistence) as the average distance in unconstrained ordination space of points within the foraging range of kittiwakes from the colony centroid.

| Colony | Mean Heterogeneity | ± Standard Error |
| --- | --- | --- |
| Bardsey | 1.93 | 0.08 |
| Bempton | 1.73 | 0.06 |
| Colonsay | 2.10 | 0.03 |
| Copinsay | 2.37 | 0.05 |
| Coquet | 1.16 | 0.06 |
| Filey | 1.73 | 0.03 |
| Fowlsheugh | 1.42 | 0.06 |
| Isle Of May | 1.17 | 0.04 |
| Lambay | 1.78 | 0.08 |
| Muckle Skerry | 1.36 | 0.04 |
| Puffin Island | 1.79 | 0.05 |
| Rathlin | 1.98 | 0.05 |
| Skomer | 1.68 | 0.09 |
| St Martins | 1.39 | 0.05 |
| Whinnyfold | 1.19 | 0.07 |


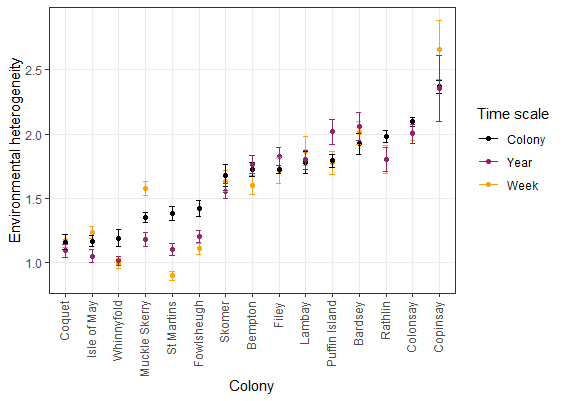


Figure A1. Mean value of environmental heterogeneity from Principal coordinate analysis at all study colonies according to the temporal resolution of calculation: Colony = colony-mean environmental heterogeneity used in all analyses presented in the main paper, Year = annual measure of environmental heterogeneity for 2017 used alongside other study years (2010-6) to calculate colony-mean heterogeneity, and Week = environmental heterogeneity calculated for a single week (June 18-26, 2017) at all colonies. Colonies ordered by increasing colony-mean heterogeneity from left to right on the x axis, error bars show standard error. We found no effect of the temporal resolution of the environmental heterogeneity measure (colony-mean/year/week) on the value of environmental heterogeneity, using a linear regression with colony as a random effect (χ ^2^_2_ = 2.09, p = 0.352).


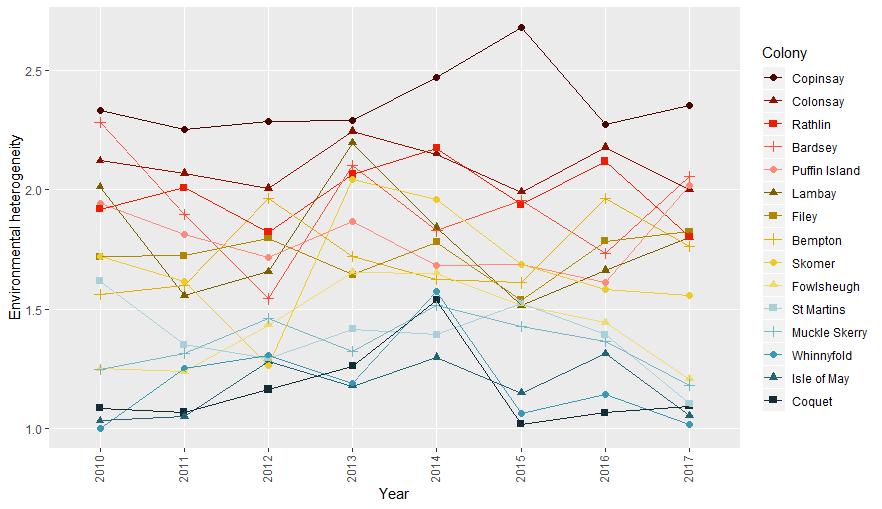


Figure A2. Value of environmental heterogeneity across study years at each colony, indicating that individual colonies were relatively consistent in their degree of environmental heterogeneity between years.


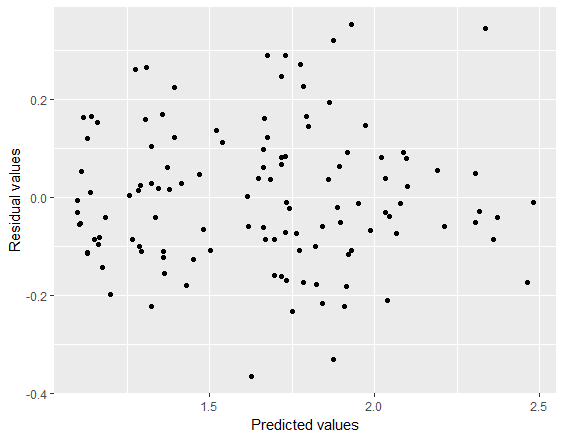


Figure A3. Residuals vs. predicted values from an ANOVA of environmental heterogeneity with colony and year. Residuals show no directional pattern, suggesting that colony and year explain the a large amount of the variance in environmental heterogeneity, and thus a single value of heterogeneity for each colony is more appropriate for analyses of the effect of heterogeneity on foraging behaviour and reproductive success.


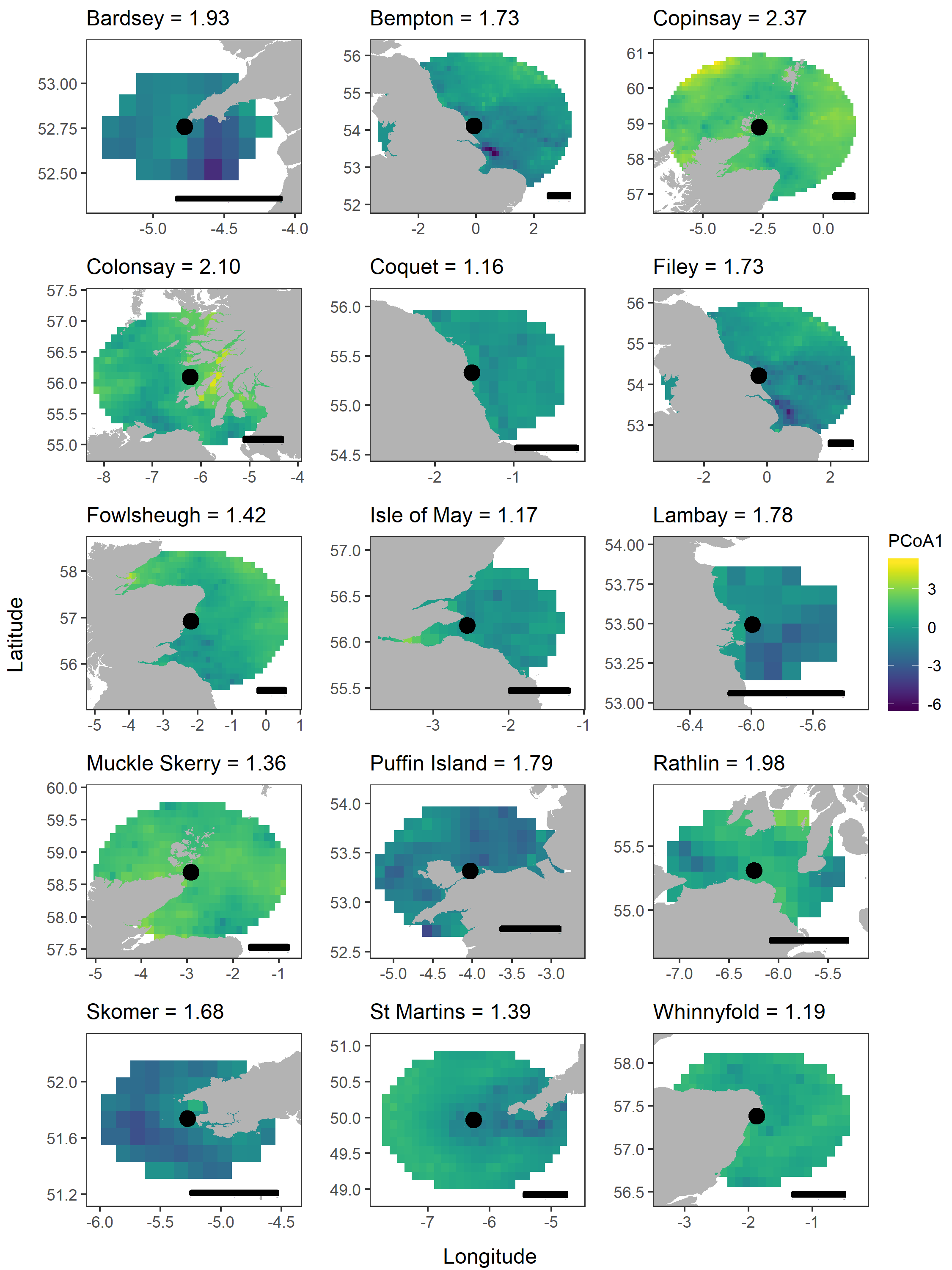


Figure A4. Environment within the foraging range of all colonies according to the position along the first axis (PCoA1) from the principal coordinate analysis used to calculate environmental heterogeneity, here for 2015 as an example. The value of environmental heterogeneity is given in each plot title, calculated as the mean distance in Euclidian space (using all PCoA axes) of all locations from the colony centroid. In all plots, the black scale bar shows 50 km.

Table A3. P-values for pairwise comparisons of environmental heterogeneity at different colonies, based on TukeyHSD 95% confidence intervals. Values in black type show a significant difference in environmental heterogeneity at p < 0.05.

|  | BAR | BEM | COP | COQ | CSY | FIL | FOW | IOM | LAM | MKS | PUF | RAT | SKO | STM |
| --- | --- | --- | --- | --- | --- | --- | --- | --- | --- | --- | --- | --- | --- | --- |
| BEM | 0.424 |  |  |  |  |  |  |  |  |  |  |  |  |  |
| COP | <0.001 | <0.001 |  |  |  |  |  |  |  |  |  |  |  |  |
| COQ | <0.001 | <0.001 | <0.001 |  |  |  |  |  |  |  |  |  |  |  |
| CSY | 0.679 | 0.001 | 0.050 | <0.001 |  |  |  |  |  |  |  |  |  |  |
| FIL | 0.426 | 1.000 | <0.001 | <0.001 | 0.001 |  |  |  |  |  |  |  |  |  |
| FOW | <0.001 | 0.015 | <0.001 | 0.071 | <0.001 | 0.015 |  |  |  |  |  |  |  |  |
| IOM | <0.001 | <0.001 | <0.001 | 1.000 | <0.001 | <0.001 | 0.092 |  |  |  |  |  |  |  |
| LAM | 0.871 | 1.000 | <0.001 | <0.001 | 0.009 | 1.000 | 0.001 | <0.001 |  |  |  |  |  |  |
| MKS | <0.001 | 0.001 | <0.001 | 0.468 | <0.001 | 0.001 | 1.000 | 0.538 | <0.001 |  |  |  |  |  |
| PUF | 0.932 | 1.000 | <0.001 | <0.001 | 0.015 | 1.000 | 0.001 | <0.001 | 1.000 | <0.001 |  |  |  |  |
| RAT | 1.000 | 0.091 | <0.001 | <0.001 | 0.979 | 0.092 | <0.001 | <0.001 | 0.402 | <0.001 | 0.515 |  |  |  |
| SKO | 0.121 | 1.000 | <0.001 | <0.001 | <0.001 | 1.000 | 0.092 | <0.001 | 0.993 | 0.006 | 0.979 | 0.015 |  |  |
| STM | <0.001 | 0.003 | <0.001 | 0.224 | <0.001 | 0.003 | 1.000 | 0.274 | <0.001 | 1.000 | <0.001 | <0.001 | 0.024 |  |
| WIN | <0.001 | <0.001 | <0.001 | 1.000 | <0.001 | <0.001 | 0.193 | 1.000 | <0.001 | 0.750 | <0.001 | <0.001 | <0.001 | 0.466 |

Table A4. P-values for pairwise comparisons of environmental heterogeneity during different years, based on TukeyHSD 95% confidence intervals. Values in black type show a significant difference in environmental heterogeneity at p < 0.05.

|  | 2010 | 2011 | 2012 | 2013 | 2014 | 2015 | 2016 |
| --- | --- | --- | --- | --- | --- | --- | --- |
| 2011 | 0.928 |  |  |  |  |  |  |
| 2012 | 0.976 | 1.000 |  |  |  |  |  |
| 2013 | 0.762 | 0.111 | 0.182 |  |  |  |  |
| 2014 | 0.546 | 0.047 | 0.084 | 1.000 |  |  |  |
| 2015 | 0.998 | 0.999 | 1.000 | 0.354 | 0.189 |  |  |
| 2016 | 1.000 | 0.980 | 0.996 | 0.600 | 0.381 | 1.000 |  |
| 2017 | 0.937 | 1.000 | 1.000 | 0.119 | 0.051 | 0.999 | 0.983 |

**Appendix B. GPS tracking sample sizes & summary trip metrics**

Table B1. Sample sizes of individuals, trips and tracking dates at all colonies and years. Data are from GPS tracking of black-legged kittiwakes, in all cases whilst birds were rearing chicks.

| **Colony** | **Longitude** | **Latitude** | **Year** | **No. of Individuals** | **No. of trips** | **Mean trips per individual** | **Unique tracking days** | **First tracking date** | **Last tracking date** | **Individuals with one trip** | **Individuals with 2-3 trips** | **Individuals with >4 trips** |
| --- | --- | --- | --- | --- | --- | --- | --- | --- | --- | --- | --- | --- |
| ALL |  |  | ALL | 415 | 1567 | 3.78 | 176 | 12/06/2010 | 09/07/2017 | 82 | 170 | 168 |
| Bardsey | -4.870 | 52.758 | 2011 | 8 | 24 | 3.00 | 8 | 04/07/2011 | 11/07/2011 | 0 | 7 | 1 |
|  |  |  | ALL | 8 | 24 | 3.00 | 8 | 04/07/2011 | 11/07/2011 | 0 | 7 | 1 |
| Bempton Cliffs | -0.077 | 54.114 | 2010 | 15 | 28 | 1.87 | 7 | 12/06/2010 | 04/07/2010 | 8 | 6 | 1 |
|  |  |  | 2011 | 7 | 16 | 2.29 | 8 | 07/06/2011 | 23/06/2011 | 4 | 1 | 2 |
|  |  |  | 2012 | 8 | 24 | 3.00 | 8 | 27/06/2012 | 04/07/2012 | 1 | 4 | 3 |
|  |  |  | 2013 | 15 | 55 | 3.67 | 5 | 29/06/2013 | 03/07/2013 | 2 | 4 | 9 |
|  |  |  | 2015 | 14 | 51 | 3.64 | 8 | 22/06/2015 | 29/06/2015 | 0 | 7 | 7 |
|  |  |  | ALL | 59 | 174 | 2.95 | 36 | 12/06/2010 | 29/06/2015 | 15 | 22 | 22 |
| Copinsay | -2.666 | 58.900 | 2010 | 12 | 32 | 2.67 | 8 | 13/06/2010 | 13/07/2010 | 5 | 4 | 3 |
|  |  |  | 2011 | 7 | 21 | 3.00 | 5 | 02/07/2011 | 06/07/2011 | 2 | 3 | 2 |
|  |  |  | 2012 | 7 | 34 | 4.86 | 6 | 26/06/2012 | 17/07/2012 | 1 | 1 | 5 |
|  |  |  | ALL | 26 | 87 | 3.35 | 19 | 13/06/2010 | 17/07/2012 | 8 | 8 | 10 |
| Coquet | -1.537 | 55.307 | 2011 | 13 | 32 | 2.46 | 4 | 14/06/2011 | 17/06/2011 | 3 | 8 | 2 |
|  |  |  | 2012 | 13 | 60 | 4.62 | 12 | 17/06/2012 | 06/07/2012 | 1 | 2 | 10 |
|  |  |  | ALL | 26 | 92 | 3.54 | 16 | 14/06/2011 | 06/07/2012 | 4 | 10 | 12 |
| Colonsay | -6.238 | 56.089 | 2010 | 8 | 21 | 2.63 | 11 | 26/06/2010 | 14/07/2010 | 3 | 2 | 3 |
|  |  |  | 2011 | 21 | 30 | 1.43 | 24 | 27/06/2011 | 24/07/2011 | 15 | 5 | 1 |
|  |  |  | 2012 | 20 | 37 | 1.85 | 17 | 02/07/2012 | 21/07/2012 | 13 | 4 | 3 |
|  |  |  | 2013 | 8 | 22 | 2.75 | 15 | 04/07/2013 | 21/07/2013 | 2 | 4 | 2 |
|  |  |  | 2014 | 12 | 38 | 3.17 | 15 | 30/06/2014 | 21/07/2014 | 1 | 6 | 5 |
|  |  |  | ALL | 69 | 148 | 2.14 | 82 | 26/06/2010 | 21/07/2014 | 34 | 21 | 14 |
| Filey | -0.272 | 54.219 | 2013 | 14 | 44 | 3.14 | 5 | 30/06/2013 | 04/07/2013 | 2 | 8 | 4 |
|  |  |  | 2015 | 12 | 44 | 3.67 | 8 | 22/06/2015 | 29/06/2015 | 0 | 8 | 4 |
|  |  |  | ALL | 26 | 88 | 3.38 | 13 | 30/06/2013 | 29/06/2015 | 2 | 16 | 8 |
| Fowlsheugh | -2.197 | 56.925 | 2012 | 13 | 26 | 2.00 | 8 | 19/06/2012 | 26/06/2012 | 4 | 8 | 1 |
|  |  |  | ALL | 13 | 26 | 2.00 | 8 | 19/06/2012 | 26/06/2012 | 4 | 8 | 1 |
| Isle of May | -2.553 | 56.181 | 2013 | 16 | 42 | 2.63 | 9 | 25/06/2013 | 14/07/2013 | 3 | 11 | 2 |
|  |  |  | ALL | 16 | 42 | 2.63 | 9 | 25/06/2013 | 14/07/2013 | 3 | 11 | 2 |
| Lambay | -5.997 | 53.495 | 2010 | 10 | 25 | 2.50 | 4 | 14/07/2010 | 19/07/2010 | 0 | 9 | 1 |
|  |  |  | ALL | 10 | 25 | 2.50 | 4 | 14/07/2010 | 19/07/2010 | 0 | 9 | 1 |
| Muckle Skerry | -2.917 | 58.689 | 2012 | 7 | 40 | 5.71 | 6 | 19/06/2012 | 05/07/2012 | 0 | 1 | 6 |
|  |  |  | 2013 | 7 | 40 | 5.71 | 4 | 12/07/2013 | 15/07/2013 | 1 | 1 | 5 |
|  |  |  | 2014 | 12 | 115 | 9.58 | 3 | 02/07/2014 | 04/07/2014 | 0 | 0 | 11 |
|  |  |  | ALL | 26 | 195 | 7.50 | 13 | 19/06/2012 | 04/07/2014 | 1 | 2 | 22 |
| Puffin Island | -4.026 | 53.321 | 2010 | 14 | 133 | 9.50 | 11 | 28/06/2010 | 14/07/2010 | 0 | 0 | 13 |
|  |  |  | 2011 | 20 | 116 | 5.80 | 15 | 27/06/2011 | 11/07/2011 | 1 | 4 | 15 |
|  |  |  | 2012 | 14 | 70 | 5.00 | 12 | 26/06/2012 | 12/07/2012 | 0 | 4 | 10 |
|  |  |  | 2015 | 9 | 41 | 4.56 | 3 | 10/07/2015 | 12/07/2015 | 0 | 4 | 5 |
|  |  |  | 2016 | 10 | 51 | 5.10 | 4 | 28/06/2016 | 01/07/2016 | 1 | 4 | 5 |
|  |  |  | ALL | 63 | 411 | 6.52 | 45 | 28/06/2010 | 01/07/2016 | 2 | 16 | 48 |
| Rathlin | -6.265 | 55.307 | 2017 | 17 | 83 | 4.88 | 4 | 06/07/2017 | 09/07/2017 | 0 | 4 | 13 |
|  |  |  | ALL | 17 | 83 | 4.88 | 4 | 06/07/2017 | 09/07/2017 | 0 | 4 | 13 |
| Skomer | -5.275 | 51.738 | 2016 | 11 | 20 | 1.82 | 2 | 27/06/2016 | 28/06/2016 | 4 | 7 | 0 |
|  |  |  | 2017 | 6 | 13 | 2.17 | 3 | 27/06/2017 | 29/06/2017 | 2 | 3 | 1 |
|  |  |  | ALL | 14 | 33 | 2.36 | 5 | 27/06/2016 | 29/06/2017 | 6 | 10 | 1 |
| St Martins | -6.262 | 49.966 | 2010 | 15 | 49 | 3.27 | 10 | 19/06/2010 | 18/07/2010 | 0 | 11 | 4 |
|  |  |  | 2011 | 13 | 36 | 2.77 | 10 | 16/06/2011 | 29/06/2011 | 1 | 9 | 3 |
|  |  |  | ALL | 28 | 85 | 3.04 | 20 | 19/06/2010 | 29/06/2011 | 1 | 20 | 7 |
| Whinnyfold | -1.869 | 57.430 | 2012 | 14 | 54 | 3.86 | 14 | 11/06/2012 | 08/07/2012 | 2 | 6 | 6 |
|  |  |  | ALL | 14 | 54 | 3.86 | 14 | 11/06/2012 | 08/07/2012 | 2 | 6 | 6 |

Table B2. Summary characteristics from foraging trips of black-legged kittiwakes tracked using GPS loggers. Foraging trips were defined when points were > 500 m from the colony and trips were > 14 minute duration.

| **Colony** | **Year** | **Mean trip duration (hrs)** | **± se** | **Trip duration range (hrs)** | **Mean total distance travelled (km)** | **± se** | **Total distance travelled range (km)** | **Mean Max distance to colony (km)** | **± se** | **Max distance to colony range (km)** |
| --- | --- | --- | --- | --- | --- | --- | --- | --- | --- | --- |
| ALL | ALL | 6.49 | 0.26 | 0 - 96.3 | 76.85 | 2.96 | 0.1 - 1189.4 | 23.34 | 0.84 | 0.3 - 229.7 |
| Bardsey | 2011 | 4.38 | 0.64 | 0.3 - 12.9 | 69.04 | 9.44 | 1.1 - 188.6 | 18.99 | 2.16 | 0.6 - 37.7 |
|  | ALL | 4.38 | 0.64 | 0.3 - 12.9 | 69.04 | 9.44 | 1.1 - 188.6 | 18.99 | 2.16 | 0.6 - 37.7 |
| Bempton Cliffs | 2010 | 4.55 | 0.84 | 0.1 - 18.8 | 82.06 | 15.35 | 0.6 - 284.6 | 35.25 | 6.78 | 0.5 - 121.4 |
|  | 2011 | 11.29 | 3.23 | 0.4 - 46.8 | 99.49 | 31.48 | 1.8 - 382.4 | 32.04 | 10.03 | 0.8 - 137.6 |
|  | 2012 | 14.89 | 3.05 | 0.3 - 58.4 | 214.49 | 42.01 | 1.1 - 695.9 | 82.39 | 15.14 | 0.9 - 220.7 |
|  | 2013 | 2.72 | 0.44 | 0 - 14.7 | 43.22 | 7.83 | 0.1 - 240.8 | 17.01 | 2.84 | 0.5 - 94.4 |
|  | 2015 | 10.68 | 2.21 | 0.2 - 88.1 | 162.59 | 29.39 | 1.3 - 899.5 | 53.61 | 8.54 | 0.5 - 195.4 |
|  | ALL | 7.81 | 0.91 | 0 - 88.1 | 113.26 | 12.15 | 0.1 - 899.5 | 41.07 | 3.99 | 0.5 - 220.7 |
| Copinsay | 2010 | 9.19 | 1.64 | 0.2 - 30.1 | 104.27 | 33.62 | 1 - 689 | 32.37 | 10.64 | 0.6 - 229.7 |
|  | 2011 | 5.52 | 1.41 | 0.3 - 18.6 | 48.07 | 12.87 | 1.7 - 217.3 | 14.96 | 4.48 | 0.8 - 86.6 |
|  | 2012 | 4.95 | 0.78 | 0.2 - 15.7 | 68.54 | 13.01 | 1.7 - 341.8 | 24.36 | 4.92 | 0.9 - 129.9 |
|  | ALL | 6.65 | 0.78 | 0.2 - 30.1 | 76.74 | 13.80 | 1 - 689 | 25.04 | 4.50 | 0.6 - 229.7 |
| Coquet | 2011 | 5.04 | 0.73 | 0.3 - 14.2 | 83.32 | 11.65 | 1.1 - 221.3 | 26.98 | 3.87 | 0.7 - 77.5 |
|  | 2012 | 2.76 | 0.37 | 0.4 - 17.9 | 30.30 | 4.68 | 4.7 - 244.7 | 9.03 | 1.17 | 2.3 - 47.6 |
|  | ALL | 3.55 | 0.37 | 0.3 - 17.9 | 48.74 | 5.69 | 1.1 - 244.7 | 15.27 | 1.78 | 0.7 - 77.5 |
| Colonsay | 2010 | 10.98 | 3.64 | 0.3 - 76.1 | 126.63 | 30.02 | 0.6 - 434.7 | 34.09 | 7.14 | 0.7 - 124.6 |
|  | 2011 | 28.05 | 4.45 | 0.9 - 96.3 | 201.18 | 33.76 | 1.7 - 806.2 | 35.78 | 4.03 | 1.1 - 76.2 |
|  | 2012 | 25.30 | 3.50 | 0.3 - 80.1 | 223.17 | 31.45 | 1.9 - 740.5 | 36.71 | 3.87 | 0.9 - 124.9 |
|  | 2013 | 16.48 | 3.50 | 0.5 - 53.3 | 136.59 | 29.59 | 1.5 - 513 | 30.02 | 5.68 | 1 - 73.7 |
|  | 2014 | 12.35 | 2.46 | 0 - 78.7 | 141.97 | 34.38 | 0.4 - 1189.4 | 32.11 | 4.68 | 0.5 - 97.3 |
|  | ALL | 19.19 | 1.67 | 0 - 96.3 | 171.29 | 15.13 | 0.4 - 1189.4 | 33.97 | 2.17 | 0.5 - 124.9 |
| Filey | 2013 | 7.92 | 1.36 | 0.2 - 41.6 | 146.99 | 25.91 | 0.5 - 682.9 | 51.90 | 8.39 | 0.6 - 172.9 |
|  | 2015 | 10.34 | 2.42 | 0.2 - 66.4 | 161.36 | 34.60 | 1.6 - 885.1 | 53.99 | 10.34 | 0.8 - 200.6 |
|  | ALL | 9.13 | 1.39 | 0.2 - 66.4 | 154.17 | 21.50 | 0.5 - 885.1 | 52.95 | 6.62 | 0.6 - 200.6 |
| Fowlsheugh | 2012 | 11.89 | 1.63 | 0.4 - 31 | 200.96 | 24.23 | 6.1 - 448.6 | 74.40 | 8.31 | 3.1 - 173 |
|  | ALL | 11.89 | 1.63 | 0.4 - 31 | 200.96 | 24.23 | 6.1 - 448.6 | 74.40 | 8.31 | 3.1 - 173 |
| Isle of May | 2013 | 9.13 | 1.36 | 0.3 - 52 | 89.68 | 12.29 | 0.6 - 386.3 | 29.50 | 3.64 | 0.7 - 78.7 |
|  | ALL | 9.13 | 1.36 | 0.3 - 52 | 89.68 | 12.29 | 0.6 - 386.3 | 29.50 | 3.64 | 0.7 - 78.7 |
| Lambay | 2010 | 4.05 | 0.64 | 0.3 - 12.9 | 50.03 | 6.56 | 1 - 122.8 | 19.22 | 2.29 | 0.7 - 39.6 |
|  | ALL | 4.05 | 0.64 | 0.3 - 12.9 | 50.03 | 6.56 | 1 - 122.8 | 19.22 | 2.29 | 0.7 - 39.6 |
| Muckle Skerry | 2012 | 3.16 | 0.58 | 0.3 - 21.4 | 50.94 | 10.11 | 3.3 - 323 | 14.44 | 3.60 | 1.5 - 121.7 |
|  | 2013 | 1.98 | 0.32 | 0.3 - 7.7 | 23.28 | 4.13 | 1 - 91.7 | 7.73 | 1.28 | 0.7 - 31.1 |
|  | 2014 | 1.17 | 0.12 | 0.2 - 7.6 | 13.79 | 1.63 | 1.2 - 120.3 | 4.84 | 0.63 | 0.7 - 47.3 |
|  | ALL | 1.74 | 0.16 | 0.2 - 21.4 | 23.35 | 2.63 | 1 - 323 | 7.40 | 0.90 | 0.7 - 121.7 |
| Puffin Island | 2010 | 2.58 | 0.22 | 0.2 - 12.8 | 29.14 | 2.70 | 0.7 - 182.1 | 9.07 | 0.95 | 0.3 - 71 |
|  | 2011 | 5.00 | 0.44 | 0.2 - 23.6 | 58.23 | 4.58 | 0.7 - 245.2 | 17.78 | 1.25 | 0.3 - 61.6 |
|  | 2012 | 4.94 | 0.86 | 0.2 - 52 | 67.41 | 8.32 | 0.7 - 331.6 | 21.44 | 2.50 | 0.8 - 78.1 |
|  | 2015 | 5.48 | 1.50 | 0.2 - 40.7 | 40.15 | 9.60 | 0.5 - 219.4 | 10.56 | 2.71 | 0.4 - 68.3 |
|  | 2016 | 2.69 | 0.49 | 0.1 - 13.2 | 22.46 | 5.00 | 0.1 - 169 | 7.27 | 1.73 | 0.3 - 53.7 |
|  | ALL | 3.97 | 0.27 | 0.1 - 52 | 44.14 | 2.52 | 0.1 - 331.6 | 13.56 | 0.76 | 0.3 - 78.1 |
| Rathlin | 2017 | 3.44 | 0.31 | 0.2 - 14.7 | 39.67 | 3.70 | 0.4 - 167.1 | 13.49 | 1.29 | 0.3 - 57.1 |
|  | ALL | 3.44 | 0.31 | 0.2 - 14.7 | 39.67 | 3.70 | 0.4 - 167.1 | 13.49 | 1.29 | 0.3 - 57.1 |
| Skomer | 2016 | 9.91 | 1.93 | 0.3 - 24.2 | 87.54 | 17.60 | 0.6 - 243.3 | 19.95 | 3.28 | 0.4 - 44.9 |
|  | 2017 | 9.31 | 2.54 | 0.5 - 32.5 | 93.80 | 20.88 | 2.4 - 273.7 | 25.02 | 4.20 | 1.1 - 49.2 |
|  | ALL | 9.68 | 1.52 | 0.3 - 32.5 | 90.00 | 13.27 | 0.6 - 273.7 | 21.95 | 2.58 | 0.4 - 49.2 |
| St Martins | 2010 | 3.78 | 0.39 | 0.2 - 13.6 | 61.77 | 7.64 | 0.8 - 193.6 | 20.40 | 3.04 | 0.7 - 69 |
|  | 2011 | 6.09 | 0.89 | 0.3 - 23.7 | 74.38 | 16.53 | 0.9 - 332.9 | 23.02 | 5.25 | 0.7 - 110.5 |
|  | ALL | 4.76 | 0.45 | 0.2 - 23.7 | 67.11 | 8.24 | 0.8 - 332.9 | 21.51 | 2.82 | 0.7 - 110.5 |
| Whinnyfold | 2012 | 7.00 | 0.97 | 0.3 - 32.6 | 75.53 | 11.56 | 0.5 - 320.9 | 25.28 | 3.48 | 0.6 - 88.9 |
|  | ALL | 7.00 | 0.97 | 0.3 - 32.6 | 75.53 | 11.56 | 0.5 - 320.9 | 25.28 | 3.48 | 0.6 - 88.9 |

Table B3. Distribution of kittiwake tracking data (GPS) and reproductive success data (RS) across colonies and years used in this study.

| Colony | Year |  |  |  |  |  |  |  | Years with GPS data | Years with RS data | Years with both |
| --- | --- | --- | --- | --- | --- | --- | --- | --- | --- | --- | --- |
|  | 2010 | 2011 | 2012 | 2013 | 2014 | 2015 | 2016 | 2017 |  |  |  |
| Bardsey |  | GPS + RS | RS | RS | RS | RS | RS |  | 1 | 6 | 1 |
| Bempton cliffs | GPS + RS | GPS + RS | GPS + RS | GPS + RS | RS | GPS + RS | RS | RS | 5 | 8 | 5 |
| Copinsay | GPS + RS | GPS + RS | GPS + RS |  | RS |  |  |  | 3 | 3 | 3 |
| Coquet | RS | GPS + RS | GPS + RS | RS | RS | RS |  |  | 2 | 6 | 2 |
| Colonsay | GPS | GPS | GPS | GPS | GPS |  |  |  | 5 | 0 | 0 |
| Filey |  |  | RS | GPS + RS | RS | GPS + RS | RS |  | 2 | 5 | 2 |
| Fowlsheugh | RS | RS | GPS + RS | RS | RS | RS | RS |  | 1 | 7 | 1 |
| Isle of may | RS | RS | RS | GPS + RS | RS | RS | RS |  | 1 | 7 | 1 |
| Lambay | GPS + RS | RS |  |  |  |  |  |  | 1 | 2 | 1 |
| Muckle Skerry |  |  | GPS | GPS | GPS |  |  |  | 3 | 0 | 0 |
| Puffin island | GPS + RS | GPS + RS | GPS + RS | RS | RS | GPS + RS | GPS + RS |  | 5 | 7 | 5 |
| Rathlin |  |  |  |  |  |  |  | GPS | 1 | 0 | 0 |
| Skomer | RS | RS | RS | RS | RS | RS | GPS + RS | GPS | 2 | 7 | 1 |
| St martins | GPS + RS | GPS + RS |  |  |  |  |  |  | 2 | 2 | 2 |
| Whinnyfold |  |  | GPS |  |  |  |  |  | 1 | 0 | 0 |
| Colonies with GPS data | 6 | 7 | 8 | 5 | 2 | 3 | 2 | 2 |  |  |  |
| Colonies with RS data | 9 | 10 | 9 | 8 | 9 | 8 | 7 | 1 |  |  |  |
| Colonies with both | 5 | 6 | 5 | 3 | 0 | 3 | 2 | 0 |  |  |  |

Table B4. Colony reproductive success from JNCC monitoring data, as well as colony size and number of breeding pairs within the maximum foraging range of each colony from Seabird 2000 monitoring counts.

| Colony | Year | Nests monitored | Fledged chicks | Breeding success | Colony Size | No pairs within foraging range |
| --- | --- | --- | --- | --- | --- | --- |
| Bardsey | 2011 | 31 | 16 | 0.52 | 288 | 1637 |
| Bardsey | 2012 | 44 | 23 | 0.52 |  |  |
| Bardsey | 2013 | 38 | 27 | 0.71 |  |  |
| Bardsey | 2014 | 67 | 14 | 0.21 |  |  |
| Bardsey | 2015 | 62 | 52 | 0.84 |  |  |
| Bardsey | 2016 | 66 | 27 | 0.41 |  |  |
| Bempton | 2010 | 1142 | 1332 | 1.17 | NA | 67560 |
| Bempton | 2011 | 1001 | 863 | 0.86 |  |  |
| Bempton | 2012 | 898 | 710 | 0.79 |  |  |
| Bempton | 2013 | 895 | 458 | 0.51 |  |  |
| Bempton | 2014 | 906 | 709 | 0.78 |  |  |
| Bempton | 2015 | 1058 | 774 | 0.73 |  |  |
| Bempton | 2016 | 1019 | 546 | 0.54 |  |  |
| Bempton | 2017 | 900 | 527 | 0.59 |  |  |
| Colonsay | NA | NA | NA | NA | 6485 | 29843 |
| Copinsay | 2010 | 229 | 85 | 0.37 |  |  |
| Copinsay | 2011 | 64 | 12 | 0.19 |  |  |
| Copinsay | 2012 | 32 | 20 | 0.63 |  |  |
| Copinsay | 2014 | 7 | 5 | 0.71 |  |  |
| Coquet | 2010 | 30 | 39 | 1.30 | 51 | 28149 |
| Coquet | 2011 | 30 | 45 | 1.50 |  |  |
| Coquet | 2012 | 215 | 235 | 1.09 |  |  |
| Coquet | 2013 | 30 | 36 | 1.20 |  |  |
| Coquet | 2014 | 30 | 38 | 1.27 |  |  |
| Coquet | 2015 | 30 | 38 | 1.27 |  |  |
| Filey | 2012 | 241 | 55 | 0.23 | 5120 | 67410 |
| Filey | 2013 | 223 | 58 | 0.26 |  |  |
| Filey | 2014 | 255 | 114 | 0.45 |  |  |
| Filey | 2015 | 257 | 119 | 0.46 |  |  |
| Filey | 2016 | 231 | 55 | 0.24 |  |  |
| Fowlsheugh | 2010 | 328 | 304 | 0.93 | 18377 | 148714 |
| Fowlsheugh | 2011 | 423 | 567 | 1.34 |  |  |
| Fowlsheugh | 2012 | 379 | 277 | 0.73 |  |  |
| Fowlsheugh | 2013 | 370 | 241 | 0.65 |  |  |
| Fowlsheugh | 2014 | 432 | 360 | 0.83 |  |  |
| Fowlsheugh | 2015 | 394 | 545 | 1.38 |  |  |
| Fowlsheugh | 2016 | 427 | 419 | 0.98 |  |  |
| Isle of May | 2010 | 494 | 143 | 0.29 | 3639 | 31505 |
| Isle of May | 2011 | 449 | 391 | 0.87 |  |  |
| Isle of May | 2012 | 470 | 461 | 0.98 |  |  |
| Isle of May | 2013 | 351 | 144 | 0.41 |  |  |
| Isle of May | 2014 | 403 | 472 | 1.17 |  |  |
| Isle of May | 2015 | 569 | 609 | 1.07 |  |  |
| Isle of May | 2016 | 497 | 388 | 0.78 |  |  |
| Lambay | 2010 | 390 | 363 | 0.93 | 4091 | 7925 |
| Lambay | 2011 | 462 | 476 | 1.03 |  |  |
| Muckle Skerry | NA | NA | NA | NA | 219 | 149985 |
| Puffin Island | 2010 | 117 | 106 | 0.91 | 571 | 3614 |
| Puffin Island | 2011 | 57 | 79 | 1.39 |  |  |
| Puffin Island | 2012 | 61 | 47 | 0.77 |  |  |
| Puffin Island | 2013 | 65 | 0 | 0.00 |  |  |
| Puffin Island | 2014 | 71 | 5 | 0.07 |  |  |
| Puffin Island | 2015 | 67 | 61 | 0.91 |  |  |
| Puffin Island | 2016 | 55 | 54 | 0.98 |  |  |
| Rathlin | NA | NA | NA | NA | 9917 | 12126 |
| Skomer | 2010 | 662 | 467 | 0.71 | 2257 | 2813 |
| Skomer | 2011 | 702 | 380 | 0.54 |  |  |
| Skomer | 2012 | 591 | 194 | 0.33 |  |  |
| Skomer | 2013 | 394 | 160 | 0.41 |  |  |
| Skomer | 2014 | 491 | 345 | 0.70 |  |  |
| Skomer | 2015 | 416 | 319 | 0.77 |  |  |
| Skomer | 2016 | 380 | 260 | 0.68 |  |  |
| St Martins | 2010 | 76 | 54 | 0.71 | 27 | 2024 |
| St Martins | 2011 | 74 | 9 | 0.12 |  |  |
| Winnyfold | NA | NA | NA | NA | NA | 70843 |


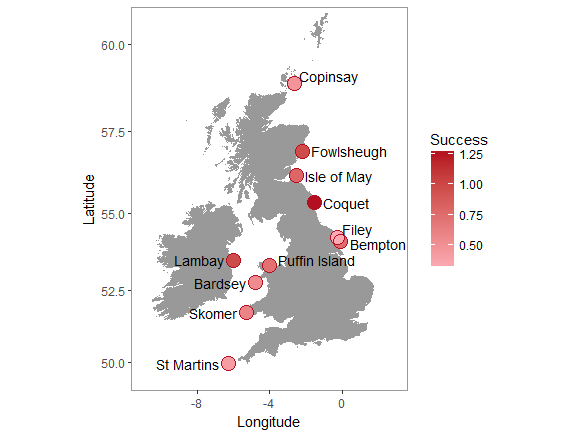


Figure B1, Map of study kittiwake colonies, for which we have reproductive success data, coloured by mean reproductive success across all years.

**Appendix C: Hidden Markov models for behavioural classification**

We used the R package moveHMM [12] for behavioural classification based on distributions of step lengths and turning angles. We used a gamma distribution to describe step lengths and a von Mises distribution to describe turning angles, and the Viterbi algorithm to estimate the most likely sequence of movement states based on the fitted hidden Markov model. We used distributions of step lengths and turning angles from previous classification of kittiwake behavioural classification to inform model starting parameters [3].

The hidden Markov model successfully classified kittiwake tracks into three movement types, which we use as proxies of behaviour: (1) resting: short step lengths and narrow turning angles (step: 0.09 ± 0.08 km; turn: μ = 0, κ = 26.36), (2) foraging: short-medium step lengths and wide turning angles (step: 0.20 ± 0.26 km; turn: μ = -0.02, κ = 0.34) & (3) transiting: long step lengths and narrow turning angles (step: 1.12 ± 0.37 km; turn: μ = 0, κ = 13.24). Distributions of step lengths and turning angles are given in Figure C1. Using the Viterbi algorithm to determine the most likely sequence of behavioural states, 16.9% of all GPS locations were classified as resting, 54.3% as foraging and 28.8% as transiting.


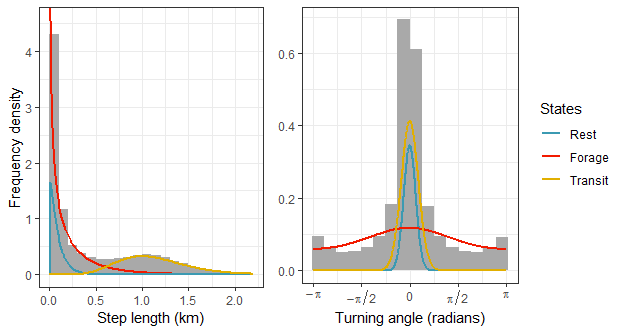


Figure C1. Histograms of observed step lengths (left) and turning angles (right) for GPS-tracked kittiwakes. Lines show fitted HMM state distributions for each behavioural state.

**Appendix D: Other environmental variables**

To verify that observed patterns were driven by environmental heterogeneity, we considered the potentially confounding effects of alternative environmental variables on foraging trip metrics and breeding success [13]. For bathymetry and stratification, which remain constant between years, we took an average value of the environment variable within the foraging range of kittiwakes at each colony. For SST and front variables, which vary temporally, we took an average of the environment variables at each colony and for each year during June-July to encompass the GPS tracking period. We used GLMMs for each trip metric in turn as the response variable, initially including year and colony as random effects to account for the nested data structure. We ran separate models for each environmental variable.

In support of results presented in the main paper, that observed changes in foraging dynamics and reproductive success can be attributed to environmental heterogeneity, we found that reproductive success was not correlated with any of the environmental variables when considered separately (Table D1). Trip duration was negatively correlated with front density (parameter estimate: -0.34, χ^2^_1_ = 4.79, p = 0.03) and front persistence (parameter estimate: -0.28, χ^2^_1_ = 4.30, p = 0.04), and total distance travelled during a foraging trip was negatively correlated with front density (parameter estimate: -0.29, χ^2^_1_ = 4.06, p = 0.04). There were no links between trip metrics and bathymetry, stratification, SST or front distance (Table D1).

Table D1. The relationship between both foraging trip metrics and reproductive success with individual environmental metrics. Significant results are shown in black type. Estimates are from GLMMs with colony as a random effect.

| **Response variable** | **Model output** | **Bathymetry** | **Stratification** | **SST** | **Front Density** | **Front persistence** | **Front distance** |
| --- | --- | --- | --- | --- | --- | --- | --- |
| Trip Duration | Test statistic | χ ^2^_1_ = 0.00 | χ ^2^_1_ = 1.28 | χ ^2^_1_ = 1.60 | χ ^2^_1_ = 4.79 | χ ^2^_1_ = 4.30 | χ ^2^_1_ = 0.12 |
|  | p-value | p = 0.995 | p = 0.258 | p = 0.205 | p = 0.029 | p = 0.038 | p = 0.729 |
|  | Estimate ± se (logged scale) | 0.00 ± 0.17 | 0.15 ± 0.14 | -0.21 ± 0.15 | -0.34 ± 0.13  R^2^ = 0.15 | -0.28 ± 0.12  R^2^ = 0.13 | 0.06 ± 0.15 |
| Total Distance | Test statistic | χ ^2^_1_ = 0.112 | χ ^2^_1_ = 1.00 | χ ^2^_1_ = 0.66 | χ ^2^_1_ = 4.06 | χ ^2^_1_ = 3.66 | χ ^2^_1_ = 0.20 |
|  | p-value | p = 0.736 | p = 0.316 | p = 0.417 | p = 0.044 | p = 0.056 | p = 0.658 |
|  | Estimate ± se (logged scale) | -0.06 ± 0.16 | 0.13 ± 0.14 | -0.14 ± 0.15 | -0.29 ± 0.13  R^2^ = 0.12 | -0.24 ± 0.12 | 0.07 ± 0.14 |
| Maximum Distance | Test statistic | χ ^2^_1_ = 0.26 | χ ^2^_1_ = 0.64 | χ ^2^_1_ = 0.40 | Χ^2^_1_ = 3.33 | χ ^2^_1_ = 3.20 | χ ^2^_1_ = 0.63 |
|  | p-value | p = 0.611 | p = 0.425 | p = 0.525 | p = 0.068 | p = 0.074 | p = 0.46 |
|  | Estimate ± se (logged scale) | -0.08 ± 0.16 | 0.10 ± 0.14 | -0.10 ± 0.14 | -0.24 ± 0.13 | -0.21 ± 0.12 | 0.11 ± 0.14 |
| Reproductive Success | Test statistic | χ ^2^_1_ = 0.46 | χ ^2^_1_ = 0.17 | χ ^2^_1_ = 0.29 | χ ^2^_1_ = 0.04 | χ ^2^_1_ = 1.07 | χ ^2^_1_ = 1.29 |
|  | p-value | p = 0.498 | p = 0.680 | p = 0.589 | p = 0.834 | p = 0.301 | p = 0.256 |
|  | Estimate ± se | -0.05 ± 0.08 | 0.03 ± 0.09 | 0.08 ± 0.06 | 0.01 ± 0.05 | 0.06 ± 0.06 | -0.07 ± 0.06 |

**Appendix E: Comparison of environmental heterogeneity with environmental variables**

There was a significant, positive correlation between bathymetry and environmental heterogeneity (F_(1,118)_ = 19.17, p < 0.001, R^2^ = 0.14, Figure E1), however this relationship was driven by high environmental heterogeneity and deep waters at Copinsay. When all years of data at Copinsay were excluded, there was no significant correlation between bathymetry and heterogeneity (F_(1,110)_ = 1.49, p = 0.225, Figure E2). There was also a significant, positive correlation between front density and heterogeneity (F_(1,118)_ = 8.49, p = 0.004), Figure E1), however the correlation explained very little variation in the data: R^2^ = 0.06. There was no correlation between environmental heterogeneity and stratification (F_(1,118)_ = 0.73, p = 0.393), sea surface temperature (F_(1,118)_ = 0.0044, p = 0.947), front persistence (F_(1,118)_ = 0.62, p = 0.433) or front distance (F_(1,118)_ = 1.30, p = 0.257). There was no relationship between environmental heterogeneity and either of the first two principal components from a PCA of mean values of environmental variables at each colony and year (PC1: F_(1,118)_ = 0.13, p = 0.719; PC2: F_(1,118)_ = 0.05, p = 0.829). In the principal component analysis, the first two principal components explained 78.1% of the total variation in mean environmental variables.


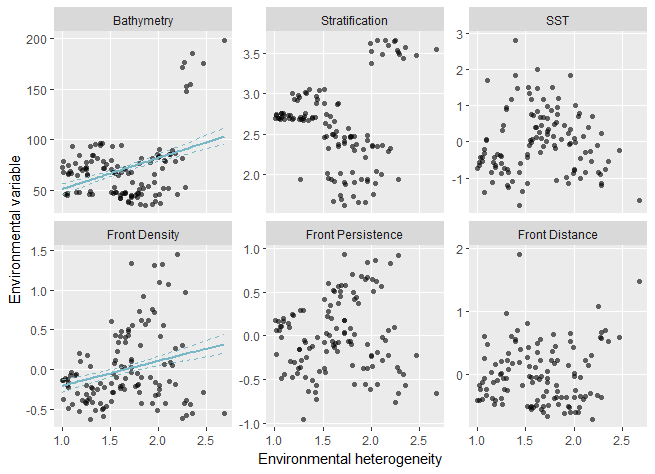


Figure E1. Comparison of environmental heterogeneity with mean values of each variable used in the principal coordinate analysis from within the foraging range of kittiwakes. Regression lines show significant correlations (± standard error) between environmental heterogeneity and bathymetry (F_(1,118)_ = 19.17, p < 0.001, R^2^ = 0.14), and front density (F_(1,118)_ = 8.49, p = 0.004, R^2^ = 0.06).


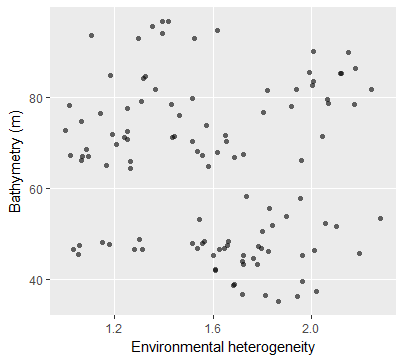


Figure E2. Comparison between environmental heterogeneity and bathymetry at all colonies, excluding Copinsay. When Copinsay is removed from the regression between bathymetry and environmental heterogeneity, there is no significant correlation (F_(1,110)_ = 1.489, p < 0.225).

**References**

1. Hijmans RJ, Etten J van. 2018 raster: Geographic analysis and modeling with raster data. *R Packag. version 2.7-15*. , 244. (doi:10.1002/asia.201100533)

2. Soanes LM, Arnould JPY, Dodd SG, Sumner MD, Green JA. 2013 How many seabirds do we need to track to define home-range area? *J. Appl. Ecol.* **50**, 671–679. (doi:10.1111/1365-2664.12069)

3. Trevail AM, Green JA, Sharples J, Polton JA, Arnould JPY, Patrick SC. 2018 Environmental heterogeneity amplifies behavioural response to a temporal cycle. *Oikos* (doi:10.1111/oik.05579)

4. Guihou K, Polton J, Harle J, Wakelin S, O’Dea E, Holt J. 2018 Kilometric Scale Modeling of the North West European Shelf Seas: Exploring the Spatial and Temporal Variability of Internal Tides. *J. Geophys. Res. Ocean.* **123**, 688–707. (doi:10.1002/2017JC012960)

5. Simpson JH, Hunter JR. 1974 Fronts in the Irish Sea. *Nature* **250**, 404–406. (doi:10.1038/250404a0)

6. Polton JA, Palmer MR, Howarth MJ. 2011 Physical and dynamical oceanography of Liverpool Bay. *Ocean Dyn.* **61**, 1421–1439. (doi:10.1007/s10236-011-0431-6)

7. Benazzouz A, Mordane S, Orbi A, Chagdali M, Hilmi K, Atillah A, Lluís Pelegrí J, Hervé D. 2014 An improved coastal upwelling index from sea surface temperature using satellite-based approach – The case of the Canary Current upwelling system. *Cont. Shelf Res.* **81**, 38–54. (doi:10.1016/J.CSR.2014.03.012)

8. Carroll G, Everett JD, Harcourt R, Slip D, Jonsen I. 2016 High sea surface temperatures driven by a strengthening current reduce foraging success by penguins. *Sci. Rep.* **6**, 22236. (doi:10.1038/srep22236)

9. Scales KL, Miller PI, Hawkes LA, Ingram SN, Sims DW, Votier SC. 2014 On the Front Line: frontal zones as priority at-sea conservation areas for mobile marine vertebrates. *J. Appl. Ecol.* **51**, 1575–1583. (doi:10.1111/1365-2664.12330)

10. Waggitt JJ, Cazenave PW, Howarth LM, Evans PGH, van der Kooij J, Hiddink JG. 2018 Combined measurements of prey availability explain habitat selection in foraging seabirds. *Biol. Lett.* **14**, 20180348. (doi:10.1098/rsbl.2018.0348)

11. Miller P. 2009 Composite front maps for improved visibility of dynamic sea-surface features on cloudy SeaWiFS and AVHRR data. *J. Mar. Syst.* **78**, 327–336. (doi:10.1016/j.jmarsys.2008.11.019)

12. Michelot T, Langrock R, Patterson TA. 2016 moveHMM: an R package for the statistical modelling of animal movement data using hidden Markov models. *Methods Ecol. Evol.* **7**, 1308–1315. (doi:10.1111/2041-210X.12578)

13. Ashmole NP. 1963 The regulation of numbers of tropical oceanic birds. *Ibis (Lond. 1859).* **103 b**, 458–473. (doi:10.1111/j.1474-919X.1963.tb06766.x)
